# *De novo* transcriptome assembly and comprehensive expression profiling in *Crocus sativus* to gain insights into apocarotenoid biosynthesis

**DOI:** 10.1038/srep22456

**Published:** 2016-03-03

**Authors:** Mukesh Jain, Prabhakar Lal Srivastava, Mohit Verma, Rajesh Ghangal, Rohini Garg

**Affiliations:** 1 Functional and Applied Genomics Laboratory, National Institute of Plant Genome Research, Aruna Asaf Ali Marg, New Delhi, India; 2 School of Computational and Integrative Sciences, Jawaharlal Nehru University, New Delhi, India

## Abstract

Saffron (*Crocus sativus* L.) is commonly known as world’s most expensive spice with rich source of apocarotenoids and possesses magnificent medicinal properties. To understand the molecular basis of apocarotenoid biosynthesis/accumulation, we performed transcriptome sequencing from five different tissues/organs of *C. sativus* using Illumina platform. After comprehensive optimization of *de novo* transcriptome assembly, a total of 105, 269 unique transcripts (average length of 1047 bp and N50 length of 1404 bp) were obtained from 206 million high-quality paired-end reads. Functional annotation led to the identification of many genes involved in various biological processes and molecular functions. In total, 54% of *C. sativus* transcripts could be functionally annotated using public databases. Transcriptome analysis of *C. sativus* revealed the presence of 16721 SSRs and 3819 transcription factor encoding transcripts. Differential expression analysis revealed preferential/specific expression of many transcripts involved in apocarotenoid biosynthesis in stigma. We have revealed the differential expression of transcripts encoding for transcription factors (MYB, MYB related, WRKY, C2C2-YABBY and bHLH) involved in secondary metabolism. Overall, these results will pave the way for understanding the molecular basis of apocarotenoid biosynthesis and other aspects of stigma development in *C. sativus*.

*Crocus sativus* L., commonly known as saffron, is a sterile triploid plant belonging to the Iridaceae (monocots) family. The plant is a perennial stemless herb widely distributed in Iran, India, Greece, Spain and Italy^1^. The *C. sativus* is world’s most expensive spice, because it is derived from the dried red stigmas of the flower, which are harvested manually and possess a distinctively pungent honey-like flavour and aroma. *C. sativus* being sterile, is vegetatively propagated by corms, which maintains its genetic characteristics, but limits genetic enhancement. The characteristic feature of the flower of *C. sativus* is the presence of three thread-like red coloured stigmas, which are dried and used in food industry as a preservative, colouring and flavouring agent. Stigmas of *C. sativus* accumulate a large amount of apocarotenoids (upto 8% of dry stigma) derived from the oxidative cleavage of zeaxanthin, crocetin and crocetin glycosides (responsible for bright natural colour), picrocrocin (responsible for bitter taste), and safranal (responsible for powerful odor)^2^. The essential oil obtained from the extract of stigmas consists of approximately 150 volatile and aromatic compounds belonging to terpenoids and flavonoids. It also contains numerous non-volatile active components, such as zeaxanthin, lycopene, and various α- and β-carotenes derived from anthocyanins and carotenoids. Maximum levels of apocarotenoids have been detected at fully-developed scarlet stage of stigma^3^,^4^. The first step in the biosynthesis of apocarotenoids involves oxidative cleavage of zeaxanthin, which produces 3-hydroxy-cyclocitral and crocetin dialdehyde, catalyzed by carotenoid cleavage dioxygenase (CCD)^5^. These two products further undergo dehydrogenation and glucosylation to form crocin and picrocrocin. After harvesting, when stigma is dried, the combined activity of heat and enzymes splits picrocrocin to yield safranal (carboxaldehyde), which represents the major component of the essential oil constituting ~70% of total volatile fraction and is responsible for distinctive aroma^6^.

*C. sativus* has been regarded as highly valued medicinal plant in Ayurveda to treat various ailments, such as depression, cold, cardiovascular diseases, nervous disorders, anxiety, and as an anti-inflammatory agent. Recently, it has been reported that safranal has antioxidant and anticonvulsant activities, relaxant and anti-depressant properties, and cytotoxic effect on cancer cells^7^. Alpha crocin was found to have antioxidant, anxiolytic and anti-cancerous properties. These apocarotenoids can be used as potential biomolecules for cancer therapy^8^,^9^,^10^,^11^. Very high market value, limited production and increasing demand for various purposes lead to frequent adulteration of *C. sativus* with yellow stamen of saffron, pomegranate fibre, red dyed silk fibre, and flowers of safflower, marigold, arnica and tinted grasses for reducing the cost of *C. sativus*. The adulterated *C. sativus* may become useless or even harmful when used for therapeutic purposes^12^,^13^. DNA marker based techniques have emerged as a widely used tool for the identification/authentication of gradually increasing adulteration in various food products, spices and medicinal plants^14^,^15^.

RNA sequencing (RNA-seq) is a powerful technique for profiling the complete gene space of any organism due to high-throughput, accuracy and reproducibility^16^. In plants, with large and complex genomes, RNA-seq has accelerated the discovery of novel genes, tissue-specific expression patterns and functional analysis. A few studies have reported EST/transcript data^17^,^18^ and characterization of few enzymes, such as carotenoid cleavage dioxygenase (CCD)^19^ and glucosyltransferases (GTs)^20^ from *C. sativus*. Recently, the transcriptome of *C. sativus* has also been reported based on RNA-seq of two tissues^18^. However, a comprehensive transcriptome analyses and expression profiling from multiple tissues of *C. sativus* is still lacking. In this study, we present the transcriptome analysis of *C. sativus* in five different tissues/organs to understand the molecular basis of apocarotenoid biosynthesis and discovery of molecular markers. *De novo* transcriptome assembly was generated after comprehensive optimization and the expression profiles of all the transcripts were analyzed. Several genes showing preferential/specific expression in stigma were identified. The gene expression patterns and putative role of known enzymes involved in apocarotenoid biosynthesis were also analyzed. These data provide a comprehensive resource to understand the biology of *C. sativus* in general and apocarotenoid biosynthesis in particular.

## Results and Discussion

### RNA sequencing and *de novo* transcriptome assembly

*De novo* transcriptome analysis provides an excellent platform to generate a comprehensive resource of the gene space in an organism without whole genome sequencing and allows the discovery of novel genes, molecular markers and tissue-specific expression patterns. To generate the transcriptome of *C. sativus*, the total RNA isolated from various tissues, including corm, tepal, leaf, stigma and stamen was subjected to sequencing on Illumina platform. A total of 206, 296, 630 high-quality (after removal of low quality and primer/adapter contaminated reads) reads (ranging from 36 to 48 million for each sample) were generated (Table 1). *De novo* assembly of high-quality total reads was performed employing two approaches. In the first approach, primary assembly was generated using various softwares, such as Velvet, Oases, ABySS, SOAPdenovo, CLC Genomics Workbench and Trinity with varying (39–99) hash (*k*-mer) lengths. A comparative analysis of the assemblies obtained from these tools was performed based on various parameters as described earlier^21^. The analysis showed that the *k*-mer value giving best assembly was different for all the softwares. The assemblies generated by Velvet showed a gradual increase in N50 and average contig lengths with increasing *k*-mer length, best being at *k*-99. *k*-59 and *k*-89 generated the best transcriptome assembly for Oases and ABySS, respectively, with largest N50 and average contig lengths (Table S1). SOAPdenovo generated contigs with largest N50 and average lengths at *k*-97 and *k*-95, respectively. The best assembly output for each tool was selected based upon the average contig length, N50 contig length and number of contigs generated (Table S2). Based on the assembly statistics, Oases was found to be the best among all the assemblers employed in this study, which generated 112,037 transcripts with largest average contig length (652 bp) and N50 length (1031 bp) at *k*-59 (Table S2).

To obtain an optimal assembly, a second approach was used employing two-step strategy (additive *k*-mer followed by assembly using TGICL). In the first step, contigs generated from all the *k*-mers of respective assembler were merged and non-redundant contigs were obtained using CD-HIT tool (Table S3). In the second step, non-redundant contigs were assembled using TGICL suite assembler. It has been reported that additive multiple *k*-mer assembly method is a better approach than single best *k*-mer to obtain better transcript diversity and contiguity^22^. The additive *k*-mer assembly approach increased the average and N50 contig lengths to a greater extent for all the assemblers (Table 2). Among all the assemblers, ABySS resulted in best assembly by generating 105, 269 unique contigs (≥200 bp) with significantly larger average contig length of 1047 bp and N50 of 1404 bp. Largest number of contigs generated via ABySS assembly showed significant similarity with rice (57131) and Arabidopsis (53925) proteins (Table 2).

**Figure 1:**
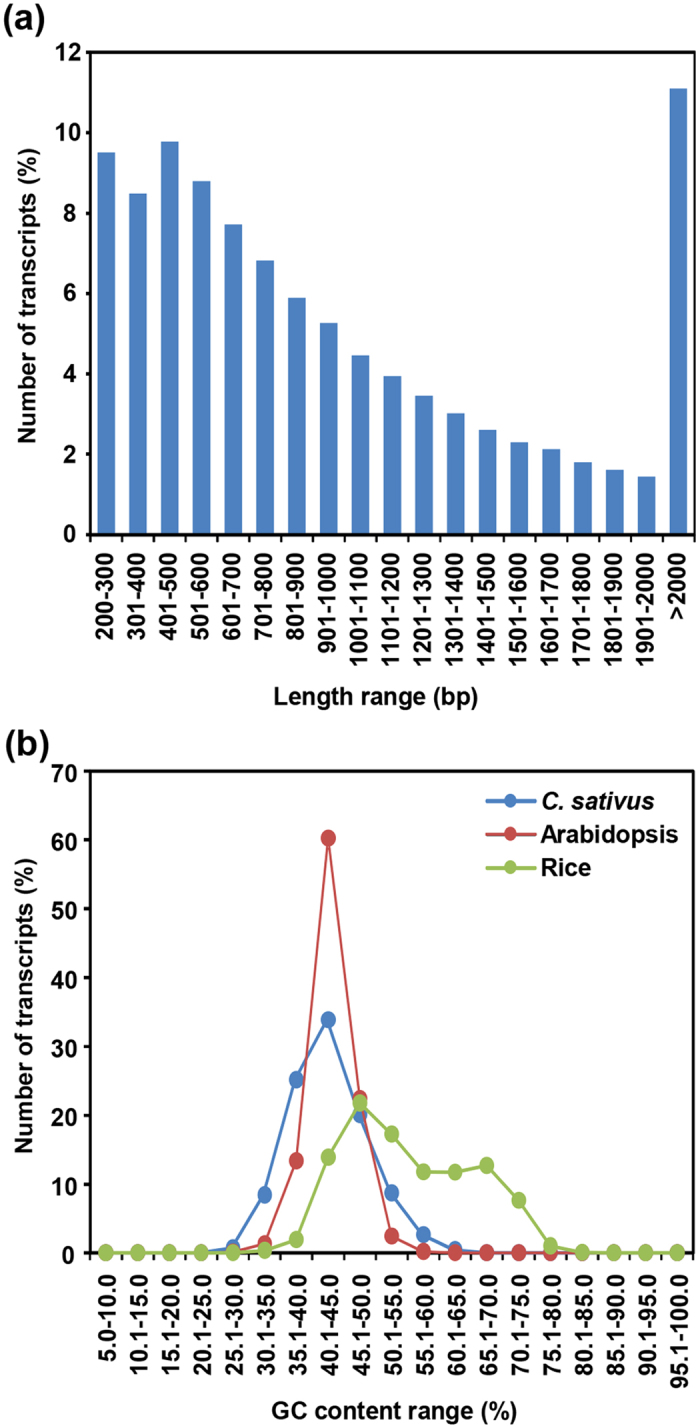
Length and GC content distribution of *C. sativus* transcripts. **(a)** Length distribution of *C. sativus* transcripts. **(b)** GC content distribution of *C. sativus* transcripts in comparison with Arabidopsis and rice.

The unique contigs obtained after *de novo* assembly were designated as *Crocus sativus* tentative consensus (CsTc) transcripts and assigned with unique identifier number from CsTc000001 to CsTc105269. The total size of the assembly was ~110 Mb with ~72% (76054) of transcripts larger than 500 bp and ~11% (11677) larger than 2 kb (Fig. 1a). The *de novo* transcriptome assembly of *C. sativus* generated in our study was better as compared to that of reported earlier^18^ with average transcript length being larger by 1.7 times and N50 length larger by 1.9 times. The average GC content (43%) was found to be quite similar to Arabidopsis (42.5%), but significantly different from rice (55%). Although the average GC contents of *C. sativus* and Arabidopsis were comparable, *C. sativus* had lower number of transcripts (34%) than that of Arabidopsis (60%) with GC content of 43% (Fig. 1b). A similarity search with the available proteomes of different plant genomes showed that largest number of *C. sativus* transcripts exhibit similarity with rice (54%) and *Brachypodium distachyon* (54%) followed by *Glycine max* (53%) (Fig. S1a). A lesser number (43%) of *C. sativus* transcripts exhibited similarity with *Physcomitrella patens* proteins. We analyzed the similarity of *C. sativus* transcripts with the publicly available transcriptomes sequences of few medicinally important plant species too. A total of 40% transcripts of *C. sativus* exhibited significant similarity with the transcriptome of *Dioscorea villosa*, 30% with *Rauvolfia serpentine*, 27% with *Panax quinquefolius* and 27% with *Catharanthus roseus* (Fig. S1b). The whole transcriptome sequence of *C. sativus* is available at the Saffron Transcriptome web page (http://nipgr.res.in/mjain.html?page=saffron).

### Functional annotation

**Figure 2:**
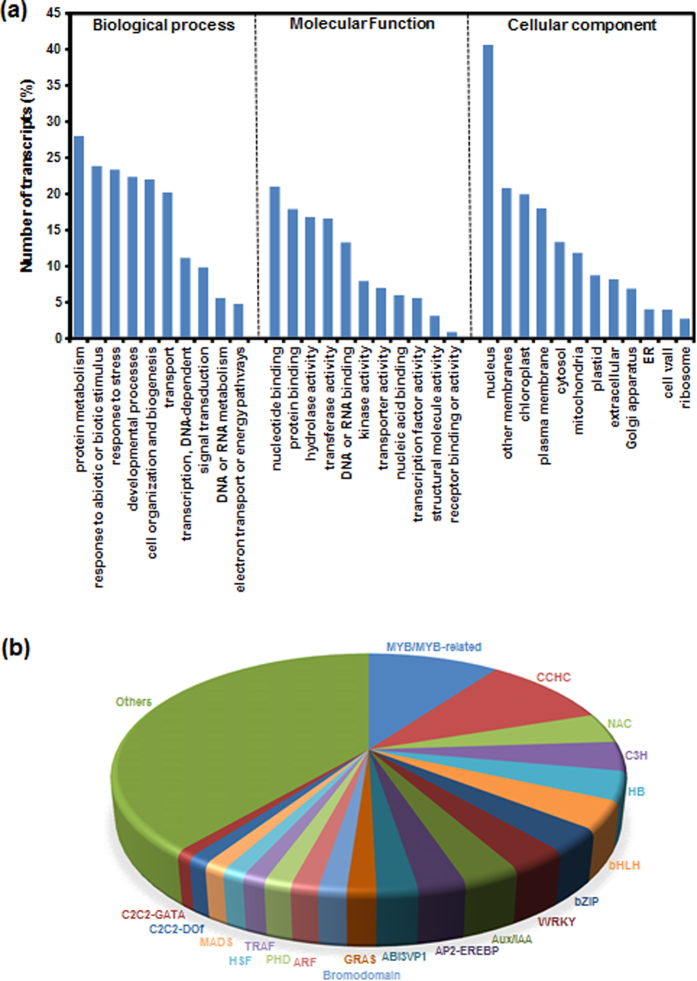
Functional annotation of *C. sativus* transcripts. **(a)** Frequency of GOSlim terms in the *C. sativus* transcripts under biological process, molecular function and cellular component categories. **(b)** Number of *C. sativus* transcripts representing different transcription factor families.

Functional annotation of the assembled transcripts can provide insights into the diverse molecular functions and biological processes represented in an organism. The functional annotation of *C. sativus* assembled transcripts was performed based on similarity with proteins/transcripts in various public databases. At least 54% of the transcripts could be functionally annotated with confidence (e-value ≤ 1e−05), whereas functions could not be assigned to rest of the transcripts, which might represent *C. sativus* specific transcripts. Further, we assigned GOSlim terms to the *C. sativus* transcripts based on the sequence similarity with Arabidopsis genes. In total, 53742 transcripts could be assigned with at least one GOSlim term under biological process (50216), molecular function (49140) and cellular component (50969) categories. Among the biological process terms, protein metabolism process (27.8%) was most represented followed by response to abiotic or biotic stimulus (23.7%), response to stress (23.2%), developmental process (22%), cell organization and biogenesis process (21.9%), and transport process (20%) (Fig. 2a). Under molecular function, the GOSlim term, nucleotide binding (20%) was most abundant followed by protein binding (16%), hydrolase activity (15%), transferase activity (15%), and DNA or RNA binding (13%) (Fig. 2a). In cellular component category, nucleus (40%) followed by other membranes (17%), chloroplast (17%) and plasma membrane (16%) were most abundant (Fig. 2a). These observations suggest that genes encoding for diverse structural, regulatory and stress-related proteins are represented in *C. sativus*. Further, the transcript sequences of *C. sativus* were searched against the KOG (euKaryotic Orthologous Groups) database for functional prediction and classification. At least 10% transcripts were assigned into 25 different KOG categories. The cluster for general function represented the largest group (17%) among all KOG classes followed by posttranslational modification, protein turnover, chaperones (10%), signal transduction mechanism (8.5%), intracellular trafficking, secretion and vascular transport (5%) and secondary metabolite biosynthesis, transport and catabolism (3%) (Fig. S2). However, 5% of the transcripts were assigned into cluster of unknown function and 1% of the transcripts were involved in defense mechanism. The putative function, GOSlim categories and KOG class assigned to the *C. sativus* transcripts are provided at the Saffron Transcriptome web page.

Transcription factors (TFs) are represented by multi-gene families and play a key regulatory role via controlling the expression of single or multiple genes through specific binding to the *cis*-regulatory elements present in the promoter regions. The number of genes encoding for different TF families varies in different plants to perform species-/tissue-specific or developmental stage specific function(s)^23^. We identified at least 3819 (3.6% of total) putative TF encoding transcripts belonging to 87 different families in the *C. sativus* transcriptome. The total number of unique transcripts encoding for TFs was substantially higher than earlier report (2601)^18^. In addition, a larger number of TF families were represented in our transcriptome data as compared to that reported by Baba and colleagues^18^. The members of MYB/MYB-related transcription factor family were found to be the most abundant (362, 9.5%) followed by CCHC (358, 9.4%), C3H (146, 3.8%), HB (142, 3.7%), bHLH (138, 3.6%), bZIP (114, 2.9%), WRKY (112, 2.9%) and Aux/IAA (111, 2.9%) (Fig. 2b). The members of TF families, including MYB, MYB-related, AP2-EREBP, WRKY and bHLH, known to regulate secondary metabolism biosynthesis in plants^24^,^25^,^26^, were present in the *C. sativus* transcriptome. These TFs might regulate the secondary metabolite (apocarotenoid) biosynthesis and accumulation in *C. sativus* in a spatio-temporal manner^23^,^27^.

### Identification of simple sequence repeats (SSRs)

**Figure 3:**
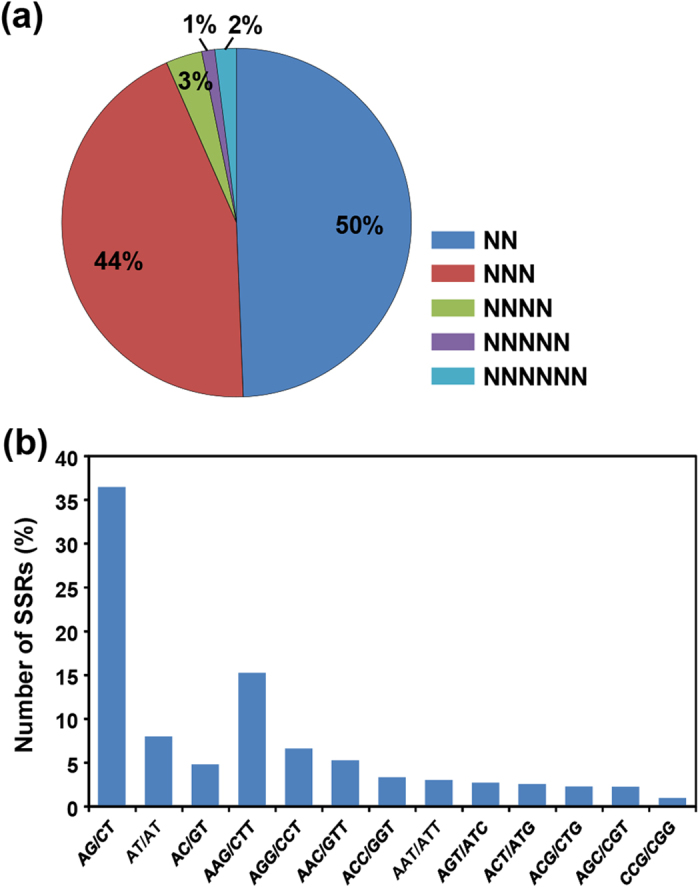
Simple sequence repeats (SSRs) in *C. sativus* transcriptome. **(a)** Distribution of SSRs in different classes. **(b)** Frequency of most abundant SSRs motifs.

SSRs have been extensively used as molecular markers for determining genetic variations across species due to their abundance, ease of development and high degree of polymorphism^28^. Many studies have been undertaken to investigate variability in *C. sativus* cultivars from different countries. A relationship between *C. sativus* and diploid autumn-flowering *Crocus* species has been established using various molecular markers, such as RAPD^29^, ISSR^30^, SSRs^31^ and SCAR^32^. The analysis of SSRs has also emerged as a popular tool for identification and authentication of different food products, spices and medicinal plants to prevent their adulteration. To identify SSRs in the *C. sativus* transcriptome, all the assembled transcripts were screened using MISA search tool. A total of 16,721 SSRs (2–6 nt) of minimum length of 12 bp were identified in 13407 (12.7%) transcripts with frequency of one SSR per 6.6 kb. However, 2576 transcripts were found to harbor more than one SSR. The identified SSRs were dominated by di-nucleotide and tri-nucleotide repeats representing about 50% (8258) and 44% (7366), respectively, of the total SSRs (Fig. 3a). Among the di-nucleotide repeats, AG/CT showed highest (36.5%) frequency followed by AT/AT (7.8%) and AC/GT (4.8%). In case of tri-nucleotide repeats, AAG/CTT showed highest occurrence (15%) followed by AGG/CCT (6.6%) and AAC/GTT (5%) along with CCG/CGG being the least abundant (0.9%) (Fig. 3b). A very small fraction of tetra- (3%), penta- (1%) and hexa-nucleotide (2%) SSRs were also identified in the *C. sativus* transcriptome (Fig. 3a). All the identified SSRs in the *C. sativus* transcripts are available at the Saffron Transcriptome web page. The availability of a large number of SSRs can greatly enhance large-scale genotyping studies for various applications in *C. sativus*.

### Differential gene expression analysis

**Figure 4:**
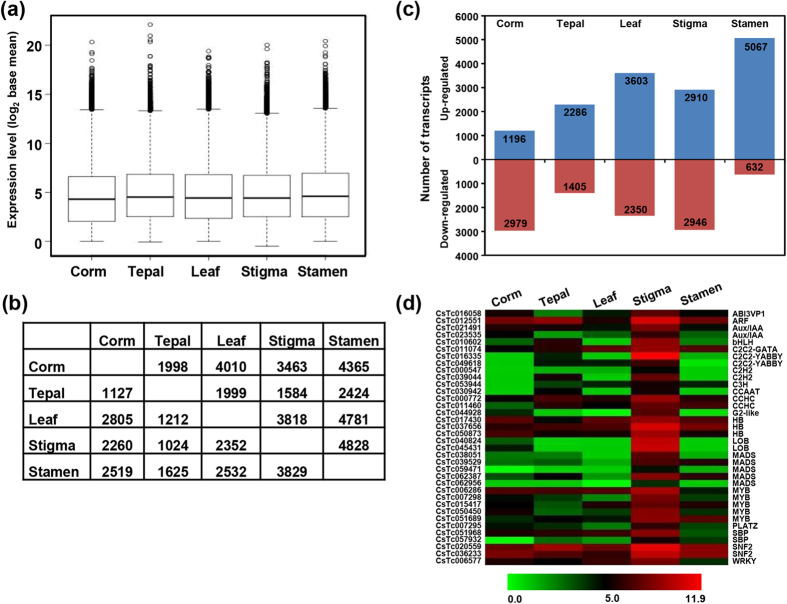
Differential expression analysis of C. sativus transcriptome. **(a)** Box plot representing the distribution of expression values of all the transcripts. **(b)** Pair-wise comparisons of different tissues to identify the differentially expressed transcripts between all the possible pairs. **(c)** The number of significantly (P-value ≤ 0.05 and at least two-fold change) up- and down-regulated transcripts in each tissue as compared to all other four tissues. **(d)** Heatmap showing expression profile of selected TFs encoding transcripts up-regulated in stigma. *C. sativus* transcript identifiers are given on the left side. Color scale representing normalized expression values is shown at the bottom.

RNA-seq has emerged as a very powerful technology to measure gene expression and tissue specificity at whole-genome level^16^,^33^. To investigate differential gene expression among different tissues, we mapped the high-quality reads from individual samples on the *C. sativus* transcriptome using CLC Genomics Workbench. Approximately 91–93% of the total reads were mapped to the *C. sativus* transcriptome (Table 1). The differentially expressed transcripts were identified among different tissues using DESeq software. The distribution of normalized expression level (log_2_ base-mean) was found to be similar in all the five tissues analyzed (Fig. 4a). The differentially expressed transcripts were identified among different tissues of *C. sativus* via pair-wise comparisons. The number of differentially expressed transcripts varied in different pairwise comparisons (Fig. 4b), suggesting substantial transcriptional differences between different tissues. Largest number of differentially expressed transcripts were detected in stamen as compared to other tissues. However, least number of transcripts showed differential expression in tepal (Fig. 4b). In stamen, at least 4828 transcripts exhibited up-regulation as compared to stigma followed by 4781 as compared to leaf. However, nearly equal number of transcripts were up-regulated in stigma as compared to stamen (3829) and leaf (3818). However, least (1024) number of transcripts were down-regulated in stigma as compared to tepal (Fig. 4b). The expression profile matrix for all the *C. sativus* transcripts is available at the Saffron Transcriptome web page.

Further, we investigated the differentially expressed transcripts in each tissue with respect to all other four tissues. The comparison of gene expression patterns in all the five tissues revealed that 2910 transcripts were up-regulated and 2946 transcripts were down-regulated in stigma. A significantly large number (5067) of transcripts were up-regulated in stamen, however, only 632 transcripts were down-regulated. A total of 3603 transcripts were up-regulated and 2350 transcripts were down-regulated in leaf, 2286 up-regulated and 1405 down-regulated in tepal, and 1196 up-regulated and 2979 down-regulated in corm (Fig. 4c). TFs are well known to play an important role in regulation of secondary metabolites biosynthesis in plants^23^,^27^,^34^. Among 2910 up-regulated transcripts in stigma, 92 transcripts were found to encode for TFs representing 37 different families. These TF encoding transcripts revealed substantial differences in their expression patterns among the five tissues with higher expression in stigma. TFs reported to be involved in secondary metabolite biosynthesis, such as Aux/IAA, MYB, MADS, C2C2-YABBY, WRKY, bHLH and SNF2 families, were also represented among the up-regulated transcripts in stigma (Fig. 4d). We further investigated the metabolic pathways in which these differentially expressed transcripts are involved (Table S4). The transcripts up-regulated in stigma mostly belonged to carbohydrate degradation, abscisic acid biosynthesis, carotenoid degradation, tetraterpenoid degradation and jasmonic acid biosynthesis. However, transcripts belonging to photosynthetic processes were significantly enriched in leaf. In corm, transcripts belonging to starch degradation, cytokinin biosynthesis, plant hormone biosynthesis and flavonoid biosynthesis were highly enriched. Phenylpropanoid derivatives biosynthesis, citrulline biosynthesis and sugars degradation pathway related transcripts were up-regulated in tepal. In stamen, transcripts belonging to superpathway of sucrose and starch metabolism, glycerol and glycerophosphodiester degradation, carbohydrates degradation, and nitroaromatic compounds degradation were highly enriched (Table S4).

**Figure 5:**
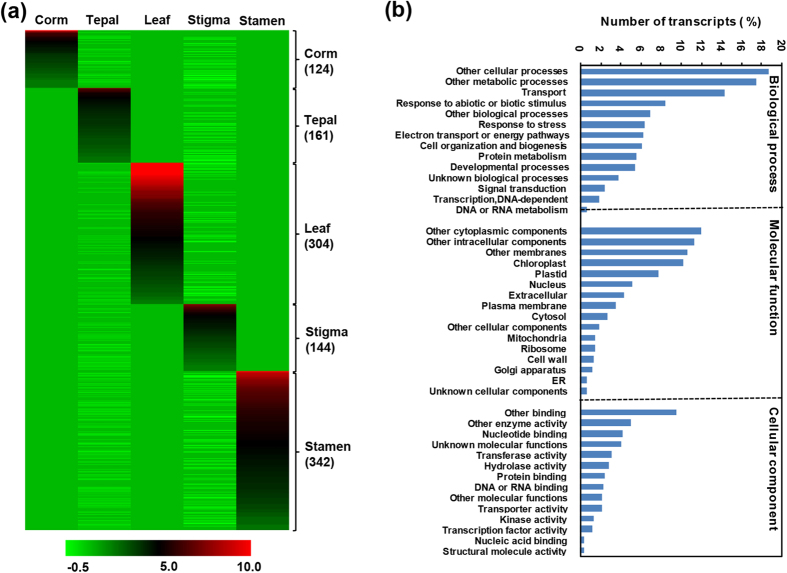
Tissue specific expression and functional annotation of *C. sativus* transcripts. **(a)** Heatmap showing tissue-specific expression in all the five tissues. Number of transcripts exhibiting specific expression in different tissues has been indicated on the right side. Color scale representing normalized expression values is shown at the bottom. **(b)** GOSlim analysis of transcripts specifically expressed in one tissue.

Further, we identified the transcripts exhibiting tissue-specific expression. A total of 1075 transcripts exhibited tissue-specific expression, 124 in corm, 161 in tepal, 304 in leaf, 144 in stigma and 342 in stamen (Fig. 5a). GOSlim analysis of these tissue-specific transcripts was performed to explore the major functional categories represented among them. In total, 270 transcripts could be assigned with at least one GOSlim term under biological process (253), molecular function (245) and cellular component (260) categories. Maximum number of transcripts belonged to other cellular process (200), metabolic process (187), transport (157), response to abiotic or biotic stimulus (90) and other biological processes (75) under biological function category (Fig. 5b). Among the molecular function categories, nucleotide binding (46), other binding (103), other enzyme activity (54) and unknown molecular functions (44) were most abundant. A large number (110) of up-regulated transcripts belonged to chloroplast under cellular component category, which is the prime site for apocarotenoid biosynthesis (Fig. 5b).

### Identification and expression analysis of candidate genes involved in apocarotenoid biosynthesis

Carotenoids are synthesized in plastids from two five-carbon building blocks, isopentenyl diphosphate (IPP) and dimethylallyl diphosphate (DMAPP), which are the products of methylerythritol phosphate (MEP) pathway. IPP and DMAPP together produce geranylgeranyl pyrophosphate (GGPP) in the presence of geranylgeranyl pyrophosphate synthase (GGDS). Phytoene synthase (PS) condenses two molecules of GGPP to produce a 40 carbon linear 15-*cis*-phytoene, which undergoes several rearrangements to produce lycopene catalyzed by phytoene desaturase (PDS) and ζ-carotene desaturase (ZDS) along with the action of two *cis*-*trans* isomerases (Z-ISO and CRTISO)^35^,^36^. Next, lycopene is converted into diverse classes of carotene backbone with one or two rings of β- or ε-type, such as α-, β- and γ-carotene catalyzed by ε-lycopene cyclase (ε-LYC)/lycopene β-cyclase (β-LYC). Lycopene β-cyclase (β-LYC) catalyzes a two-step reaction that leads to formation of β-carotene, whereas lycopene ε-cyclase and lycopene β-cyclase create one ε-ring and one β-ring to produce α-carotene, which are functionally modified with tailoring enzymes (β-carotene hydroxylase/ε-hydroxylase) to generate zeaxanthin and lutein, respectively^37^. Apocarotenoid compounds (crocetin, crocetin glycosides and picrocrocin) are derived by oxidative cleavage of zeaxanthin in several steps catalyzed by CCD, aldehyde dehydrogenase and UDP-glucosyltransferases (UGTs). Finally, picrocrocin gets converted to safranal with combined action of heat and β-glucosidase.

**Figure 6:**
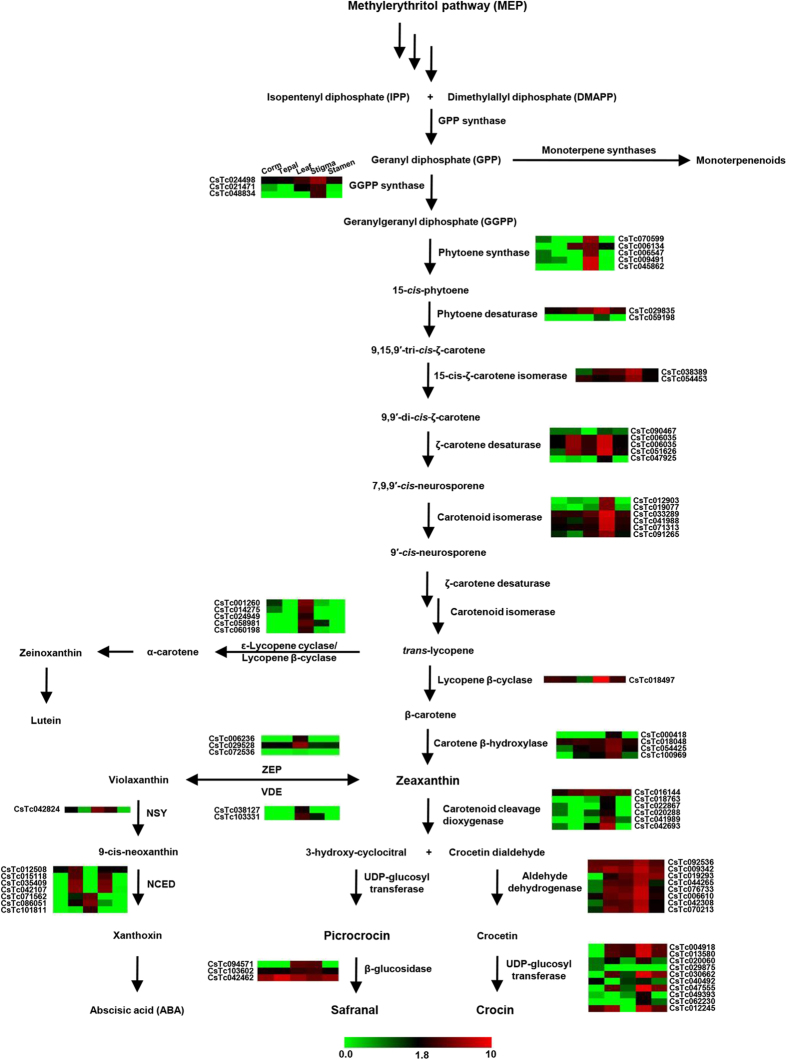
Schematic representation of apocarotenoid biosynthetic pathway and gene expression of enzyme(s) involved in *C. sativus*. The enzymes and intermediates involved in different reaction steps of apocarotenoid biosynthetic pathway known in *C. sativus* are shown. Heatmaps displaying the differential expression of transcripts encoding for enzymes (transcript identifier has been provided) involved in different steps are also shown. In the heatmaps, different columns represent tissues in order of corm, tepal, leaf, stigma and stamen from left to right (as given in the first heatmap on the pathway). Color scale representing normalized expression values is shown at the bottom.

We identified the genes encoding for all the enzymes catalyzing different intermediate reactions involved in apocarotenoid biosynthetic pathway (Fig. 6) from the *C. sativus* transcriptome. All these transcript sequences were highly conserved at the amino acid sequence level in Arabidopsis. Furthermore, we analyzed the expression patterns of all the transcripts involved in apocarotenoid biosynthetic pathway using RNA-seq data (Fig. 6). The expression analysis of transcripts involved in key enzymatic steps of apocarotenoid biosynthesis, such as carotenoid cleavage dioxygenase (CCD), glucosyltransferases, aldehyde dehydrogenases and beta glucosidases revealed their higher expression levels in stigma as compared to other tissues (Fig. 6). Similar expression patterns have been reported earlier for some of the characterized enzymes involved in apocarotenoid biosynthesis, such as carotenoid cleavage dioxygenase^19^ and glucosyltransferases^20^. These results indicate that apocarotenoids are synthesized mainly in stigma.

Further, we identified two key enzymes, zeaxanthin epoxidase (ZEP) and violaxanthin de-epoxidase (VDE), in the *C. sativus* transcriptome responsible for inter-conversion of zeaxanthin to violaxanthin and *vice versa* via antheraxanthin (epoxy-zeaxanthin) as an intermediate. It has been reported that inter-conversion of zeaxanthin and violaxanthin in higher plants is dependent on the intensity of light and associated with protective mechanism from photooxidative damage^38^,^39^. Differential expression analysis of the transcripts encoding for both the enzymes revealed their higher expression in leaf as compared to stigma (Fig. 6). The lower expression level of these transcripts in stigma can divert the zeaxanthin flux towards apocarotenoid biosynthesis. Violaxanthin is converted into 9-cis-neoxanthin catalyzed by neoxanthin synthase (NSY) and act as a precursor for abscisic acid (ABA) biosynthesis. The reaction catalyzed by neoxanthin synthase is very similar to lycopene cyclase and both of these enzymes share strong sequence similarity at amino acid level, which suggests an evolutionary relationship between them^40^. Next, we identified a key regulatory enzyme, 9-cis-epoxycarotenoid dioxygenase (NCED), implicated in abscisic acid (ABA) biosynthesis, which cleaves neoxanthin and violaxanthin to form xanthoxin, a direct precursor for ABA. The expression analysis of NCED revealed its higher expression level in tepal and stigma, which is in well agreement with the earlier report^41^. Although apocarotenoids (crocin, picrocrocin and safranal), lutein and ABA are derived from the same precursor lycopene, the transcripts encoding the enzymes involved in apocarotenoid biosynthesis were exclusively expressed in stigma, whereas the transcripts encoding for enzymes involved in lutein and ABA biosynthesis were expressed at higher level in other tissues. This could be a result of coordinated regulation of these pathways to divert the flux towards apocarotenoid biosynthesis in stigma.

It has been reported that extract of stigmas of *C. sativus* contains approximately 150 volatile and aroma yielding compounds belonging to terpenoids and flavonoids. We have identified the transcripts involved in the biosynthesis of these important compounds in the *C. sativus* transcriptome. Based on functional annotation, at least 21 transcripts belonging to terpene synthase family and 11 transcripts belonging to prenyltransferases were identified in the *C. sativus* transcriptome, which might be responsible for the accumulation of terpenoids/monoterpenoids in *C. sativus* stigma. Differential expression analysis of terpene synthase encoding transcripts revealed substantial difference in their expression pattern among all the five tissues. Many of these transcripts encoding for terpenoid biosynthesis were found to be preferentially/specifically expressed in the stigma, suggesting their biosynthesis mainly occurs in stigma (Fig. S3).

Overall, these analyses identified candidate transcripts encoding for enzymes involved in different steps of apocarotenoid biosynthesis and revealed their preferential/specific expression in stigma of *C. sativus*. Although the genes involved in different steps of apocarotenoid biosynthesis pathway were identified in the previous study^18^ as well, the availability of more number of transcripts with larger size representing these genes, and their comprehensive expression profiling in multiple tissues from our study can help better understanding of the apocarotenoid biosynthesis pathway. The functional analysis of some of these candidate transcripts can provide insights into molecular mechanisms underlying apocarotenoid biosynthetic pathway.

### Real-time PCR validation of differential expression

**Figure 7:**
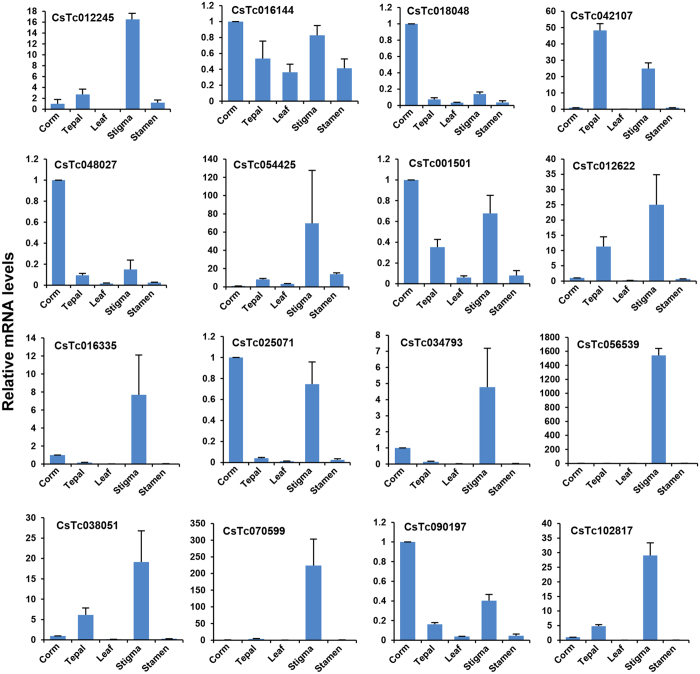
Real-time PCR validation of gene expression obtained via RNA-seq. Real-time PCR analysis of selected transcripts, including those encoding for enzymes involved in apocarotenoid biosynthesis and transcription factors, and other randomly selected transcripts exhibiting differential expression.

To validate the differential expression of selected transcripts from RNA-seq data, real-time PCR analysis was performed. The expression analysis was performed for selected genes belonging to apocarotenoid biosynthesis pathway, such as phytoene synthase (PS), beta carotene hydrolase (BCH), carotenoid cleavage dioxygenase (CCD) and glucosyltransferases (GTs) and a few TF (MADS, C2C2-YABBY and C2H2) encoding transcripts (Fig. 7). In addition, a few transcripts encoding for CYP450 mono-oxygenase, RNase H-like superfamily and ketoacyl CoA synthase showing differential expression, were also selected randomly from the transcriptome dataset and validated via real-time PCR analysis. A comparative analysis of all the selected genes revealed similar expression pattern in real-time PCR analysis as observed in RNA-seq data, suggesting the consistency in results.

In conclusion, we have generated a comprehensive transcriptome assembly representing the gene space in *C. sativus*. We identified all the transcripts encoding for enzymes involved in apocarotenoid biosynthesis/accumulation and terpenoid biosynthesis. Differential expression analysis of candidate genes along with TFs revealed that expression of most of the transcripts involved in apocarotenoid biosynthesis is restricted to stigma, which is the probable site for biosynthesis of important apocarotenoids in *C. sativus*. Furthermore, discovery of large number of SSRs in the *C. sativus* transcriptome will be useful in the marker-assisted breeding and identification/authentication of *C. sativus*. It is anticipated that these data will pave the way for elucidating the exact molecular mechanism for apocarotenoid biosynthesis in *C. sativus*.

## Methods

### Plant material

Saffron (*C. sativus* L.) plants were collected from an open farmland of a village situated in Pampore town of Pulwama district, Jammu and Kashmir, India. Different tissues, including corm, tepal, leaf, stigma and stamen were harvested from the plants and immediately frozen in liquid nitrogen and stored at −80 °C until further use.

### RNA isolation and transcriptome sequencing

Total RNA from the above tissues was isolated in three biological replicates using TRI reagent (Sigma Life Science, USA). Quantity and quality of total RNA was determined by Nanodrop spectrophotometer (Thermo Fisher Scientific) and Bioanalyzer (Agilent technologies, Singapore). Purity of total RNA was checked by estimating the absorbance ratio at 260/280 and 260/230, and RNA integrity number (RIN). The quality of total RNA isolated from corm and stigma tissues did not meet the minimum standard for Illumina sequencing. Therefore, we modified the standard protocol to obtain better quality of RNA, which included washing of RNA pellet with 5 M NaCl (2–3 times) before dissolving in RNase free water. High-quality total RNA (260/280, 1.8–2.0; 260/230 > 2.0; RIN > 7.5) pooled in equal quantity from the three biological replicates for each sample was used for transcriptome sequencing using Illumina platform to generate 100-nt long paired-end reads. To obtain high-quality clean data for *de novo* assembly, stringent quality check was performed to remove low-quality reads and adapter trimming using our in-house NGS QC Toolkit (v2.3)^42^.

### *De novo* transcriptome assembly

High-quality reads were assembled into contigs using various commonly used short-read assemblers, such as Velvet (v1.2.01)^43^, Oases (v0.2.04)^44^, ABySS (v1.2.6)^45^, SOAPdenovo (v1.04)^46^, CLC Genomics Workbench (v4.7.2) and Trinity (v2012-05-18)^47^. Transcriptome assembly was performed using two different approaches as described earlier^48^. In the first approach (best *k*-mer strategy), high-quality reads were assembled at varying *k*-mer length 39–99 using Velvet, Oases, ABySS and SOAPdenovo, whereas CLC Genomics Workbench and Trinity softwares were used at default parameters. In the second approach (additive *k*-mer followed by TGICL), a two-step strategy was employed for assembly. Firstly, contigs generated for all *k*-mers by respective assembler were merged and redundancy was removed using CD-HIT tool. Next, the non-redundant set of contigs were assembled using TGICL suite (v2.0)^49^ with minimum overlap length of 40 and maximum identity of 90. GC content analysis of *C. sativus* transcriptome was performed using in-house perl script.

### Functional annotation

To assign the putative function to each transcript of *C. sativus*, similarity search using BLASTX^50^ was performed against Arabidopsis and rice proteomes followed by NCBI non-redundant and UniRef90 databases with an *E*-value cut-off of ≤10^−5^ to find the best significant match for each transcript. GOSlim terms were assigned to each *C. sativus* transcript under molecular function, biological process and cellular component categories by comparing the sequence with Arabidopsis proteins. Likewise, classification of the *C. sativus* transcripts in different functional categories was performed using KOG database. Identification of TF families in *C. sativus* transcriptome was carried out based on hidden Markov model (HMM) profile (obtained either from the PFAM database or generated from the conserved domain alignments) search using criteria given at the Plant Transcription Factor Database (http://plntfdb.bio.uni-potsdam.de/v3.0/) as described earlier^21^.

### Identification of SSRs

*C. sativus* transcriptome was scanned for the presence of simple repeat sequences (SSRs) using MISA (MicroSAtellite) at default parameters^51^. The minimum number of repeat units for di-nucleotide was six, whereas for tri-, tetra-, penta- and hexa-nucleotide, minimum number of repeat units was more than five in the search criteria.

### Differential gene expression analysis

To estimate the expression pattern of each transcript in different tissue samples, high-quality reads from each sample were mapped on the final transcriptome assembly using CLC Genomics Workbench. A maximum of two mismatches were allowed for mapping. The read counts was normalized by calculating number of reads per kilobase per million (RPKM) for each transcript in individual tissue. Differential gene expression analysis was performed using DESeq software (v1.10.1)^52^ based on negative binomial distribution. A *P*-value cut-off of ≤ 0.05 along with at least two-fold change was used to identify significant differential expression of the transcripts. The heatmap showing tissue-specific expression patterns (log_2_ fold change) for the transcripts involved in various pathways were generated via TIGR MultiExperiment Viewer (MeV, v4.8).

### Real-time PCR analysis

For real-time PCR analysis, the gene-specific primers (Table S5) were designed using Primer Express (v3.0) software (Applied Biosystems, USA). Real-time PCRs were carried out in three independent biological replicates and three technical replicates for each biological replicate of each tissue sample as reported earlier^53^. *Ubiquitin* was used as an internal control gene for normalization.

### Data availability

The sequence data generated in this study have been deposited in the Gene Expression Omnibus under the accession number GSE65103. Transcriptome assembly, functional annotation, SSRs and gene expression data have been made available at the Saffron Transcriptome web page (http://nipgr.res.in/mjain.html? page=saffron).

## Additional Information

**How to cite this article**: Jain, M. *et al*. *De novo* transcriptome assembly and comprehensive expression profiling in *Crocus sativus* to gain insights into apocarotenoid biosynthesis. *Sci. Rep*. **6**, 22456; doi: 10.1038/srep22456 (2016).

## Supplementary Material

Supplementary Information

## Figures and Tables

**Table 1:** Summary of read data generated and mapping on the transcriptome.

Tissue sample	Raw reads	High-quality reads (%)	Mapped reads (%)
Corm	38678236	35932216 (92.90%)	32723663 (91.07%)
Tepal	53541250	48312982 (90.23%)	44807769 (92.74%)
Leaf	41354788	37650344 (91.04%)	34575420 (91.83%)
Stigma	49463790	45865500 (92.72%)	41871198 (91.29%)
Stamen	42789692	38535588 (90.06%)	35560309 (92.28%)

**Table 2:** Assembly statistics after merged assembly of the non-redundant contigs from different *k*-mers via TGICL.

	Velvet	Oases	ABySS	SOAPdenovo	CLC	Trinity
Number of contigs	191814	60426	105269	139004	65193	32996
Total size (Mb)	116.84	67.75	110.22	97.45	39.25	27.47
Minimum length (bp)	200	200	200	200	200	200
Maximum length (bp)	14710	28547	25872	17121	16323	20224
Average length (bp)	609.15	1121.28	1047.05	701.05	602.07	832.4
N50 length (bp)	808	1718	1404	975	806	1308
Contigs with significant hits in rice^1^	56758	28060	57131	50697	23630	10468
Contigs with significant hits in Arabidopsis^1^	50482	26034	53925	46417	21141	9166

^1^Number of contigs showing significant (*E*-value ≤ 1e−05) similarity with rice/Arabidopsis proteins.
