# Supplementary material for: De novo transcriptome assembly and comprehensive expression profiling in Crocus sativus to gain insights into apocarotenoid biosynthesis
Source: Sci Rep. 2016 Mar 3;6:22456. doi: 10.1038/srep22456 (PMC4776159; doi:10.1038/srep22456)

## Supplementary Information

# *De novo* transcriptome assembly and comprehensive expression profiling in *Crocus sativus* to gain insights into apocarotenoid biosynthesis

Mukesh Jain[1,2]*, Prabhakar Lal Srivastava[1], Mohit Verma[1], Rajesh Ghangal[1], Rohini Garg[1]

[1]Functional and Applied Genomics Laboratory, National Institute of Plant Genome Research, Aruna Asaf Ali Marg, New Delhi, India

[2]School of Computational and Integrative Sciences, Jawaharlal Nehru University, New Delhi, India

**Supplementary Table S1** Assembly statistics from different softwares at various k-mers.

## Velvet

| | k-39 | k-43 | k-49 | k-53 | k-59 | k-63 | k-69 | k-73 | k-79 | k-83 | k-89 | k-93 | k-99 |
|---|---|---|---|---|---|---|---|---|---|---|---|---|---|
| **Number of contigs** | 1067901 | 1005361 | 823988 | 826738 | 620163 | 508779 | 384987 | 318990 | 238632 | 191901 | 120899 | 70645 | 3276 |
| **Total size (Mb)** | 192.4 | 189.36 | 170.31 | 168.44 | 141.98 | 125.77 | 105.05 | 92.59 | 75.57 | 64.28 | 44.74 | 28.21 | 1.4 |
| **Minimum length (bp)** | 100 | 100 | 100 | 105 | 117 | 125 | 137 | 145 | 157 | 165 | 177 | 185 | 197 |
| **Maximum length (bp)** | 3823 | 3851 | 4478 | 6512 | 7689 | 7990 | 9706 | 7214 | 7877 | 7554 | 6624 | 5039 | 3113 |
| **Average length (bp)** | 180.17 | 188.35 | 206.69 | 203.74 | 228.94 | 247.21 | 272.86 | 290.25 | 316.69 | 334.94 | 370.04 | 399.27 | 426.52 |
| **N50 length (bp)** | 172 | 181 | 204 | 210 | 235 | 253 | 280 | 298 | 326 | 346 | 384 | 415 | 422 |

## Oases

| | k-39 | k-43 | k-49 | k-53 | k-59 | k-63 | k-69 | k-73 | k-79 | k-83 | k-89 | k-93 | k-99 |
|---|---|---|---|---|---|---|---|---|---|---|---|---|---|
| **Number of contigs** | 132251 | 127276 | 122163 | 118713 | 112037 | 108179 | 101107 | 94357 | 82418 | 73241 | 54885 | 38515 | 2513 |
| **Total size (Mb)** | 76.2 | 77.73 | 77.82 | 76.96 | 73.01 | 70.48 | 65.62 | 61.26 | 53.55 | 47.3 | 34.85 | 23.2 | 12.69 |
| **Minimum length (bp)** | 100 | 100 | 100 | 103 | 109 | 113 | 119 | 123 | 129 | 134 | 139 | 143 | 161 |
| **Maximum length (bp)** | 14312 | 15753 | 15602 | 15593 | 16088 | 15989 | 15989 | 15926 | 15177 | 13462 | 11732 | 7251 | 3204 |
| **Average length (bp)** | 576.17 | 610.72 | 637.04 | 648.31 | 651.69 | 651.51 | 648.97 | 649.22 | 649.74 | 645.86 | 634.97 | 602.42 | 505 |
| **N50 length (bp)** | 1014 | 1060 | 1055 | 1055 | 1031 | 994 | 944 | 918 | 882 | 844 | 779 | 695 | 498 |

## ABySS

| | k-43 | k-49 | k-53 | k-59 | k-63 | k-69 | k-73 | k-79 | k-83 | k-89 | k-93 | k-95 |
|---|---|---|---|---|---|---|---|---|---|---|---|---|
| **Number of contigs** | 457852 | 453842 | 641830 | 589368 | 538679 | 454837 | 396154 | 317209 | 266961 | 192807 | 141225 | 109747 |
| **Total size (Mb)** | 115.3 | 117.34 | 138.97 | 137.08 | 132.9 | 124.15 | 116.66 | 103.65 | 92.87 | 72.1 | 52.78 | 40.71 |
| **Minimum length (bp)** | 100 | 100 | 100 | 100 | 100 | 100 | 100 | 100 | 100 | 100 | 100 | 100 |
| **Maximum length (bp)** | 14167 | 14111 | 13966 | 11607 | 11656 | 11656 | 10848 | 10077 | 12220 | 12584 | 11040 | 6026 |
| **Average length (bp)** | 251.83 | 258.55 | 216.52 | 232.59 | 246.7 | 272.95 | 294.47 | 326.76 | 347.88 | 373.94 | 373.71 | 370.96 |
| **N50 length (bp)** | 349 | 366 | 262 | 292 | 324 | 387 | 439 | 507 | 543 | 570 | 537 | 511 |

## SOAPdenovo

| | k-43 | k-49 | k-53 | k-59 | k-63 | k-69 | k-73 | k-79 | k-83 | k-89 | k-93 | k-95 | k-97 |
|---|---|---|---|---|---|---|---|---|---|---|---|---|---|
| **Number of contigs** | 779287 | 680202 | 905609 | 757836 | 654540 | 513591 | 429756 | 319246 | 249658 | 146664 | 76258 | 41461 | 13247 |
| **Total size (Mb)** | 162.78 | 154.39 | 177.13 | 159.2 | 145.06 | 123.2 | 108.76 | 87.52 | 72.66 | 47.52 | 26.94 | 15.22 | 4.94 |
| **Minimum length (bp)** | 100 | 100 | 100 | 100 | 100 | 100 | 100 | 100 | 100 | 100 | 100 | 100 | 100 |
| **Maximum length (bp)** | 8384 | 9591 | 9591 | 9591 | 8742 | 7209 | 7209 | 7877 | 7214 | 5838 | 4980 | 3282 | 4408 |
| **Average length (bp)** | 208.88 | 226.97 | 195.59 | 210.07 | 221.62 | 239.89 | 253.08 | 274.15 | 291.05 | 323.97 | 353.23 | 367.16 | 372.78 |
| **N50 length (bp)** | 211 | 232 | 212 | 228 | 242 | 265 | 281 | 306 | 326 | 364 | 394 | 404 | 403 |

**Supplementary Table S2** Best assembly statistics from different softwares.

| | Velvet (k-93) | Oases (k-59) | ABySS (k-89) | SOAPdenovo (k-95) | CLC | Trinity |
|---|---|---|---|---|---|---|
| **Number of contigs** | 70645 | 112037 | 192807 | 41461 | 626021 | 633750 |
| **Total size (Mb)** | 28.21 | 73.01 | 72.01 | 15.22 | 169.38 | 223.42 |
| **Minimum length (bp)** | 185 | 109 | 100 | 100 | 100 | 101 |
| **Maximum length (bp)** | 5039 | 16088 | 12584 | 3282 | 16232 | 20224 |
| **Average length (bp)** | 399.27 | 651.69 | 373.94 | 367.16 | 270.56 | 352.53 |
| **N50 length (bp)** | 415 | 1031 | 570 | 404 | 294 | 578 |

**Supplementary Table S3** Assembly statistics after merging output from different k-mers followed by redundancy removal.

| | **Velvet** | **Oases** | **ABySS** | **SOAPdenovo** | **CLC** | **Trinity** |
|---|---|---|---|---|---|---|
| Number of contigs | 526076 | 207287 | 257767 | 421698 | 276991 | 224080 |
| Total size (Mb) | 206.43 | 176.90 | 194.05 | 178.72 | 115.45 | 112.33 |
| Minimum length (bp) | 200 | 200 | 200 | 200 | 200 | 200 |
| Maximum length (bp) | 9706 | 16088 | 14167 | 9591 | 16232 | 20224 |
| Average length (bp) | 392.41 | 853.40 | 752.82 | 423.83 | 427.66 | 501.31 |
| N50 length (bp) | 409 | 1358 | 953 | 453 | 455 | 595 |

**Supplementary Table S4** Significantly enriched metabolic pathways in the up-regulated transcripts in different tissues of *C. sativus*.

| **Pathway** | **P-value** |
|---|---|
| **Corm** | |
| Cytokinins-O-glucoside biosynthesis | 2.40E-07 |
| Plant Hormones Biosynthesis | 2.90E-06 |
| Cytokinins Biosynthesis | 9.8E-06 |
| Phenylpropanoid Derivatives Biosynthesis | 1.21E-05 |
| Secondary Metabolites Biosynthesis | 1.67E-05 |
| Fatty Acid Derivatives Biosynthesis | 3.77E-04 |
| Flavonoids Biosynthesis | 5.04E-04 |
| Jasmonates Biosynthesis | 0.0014 |
| Cinnamates Biosynthesis | 0.00159 |
| Superpathway of sucrose and starch metabolism II (photosynthetic tissue) | 0.00159 |
| Terpenoids Degradation | 0.01955 |
| | |
| **Leaf** | |
| Photosynthesis | 1.27E-13 |
| Chlorophyll Biosynthesis | 3.24E-06 |
| Autotrophic $CO_2$ Fixation | 2.90E-05 |
| Unusual Fatty Acid Biosynthesis | 2.03E-04 |
| Chlorophyll a-Biosynthesis | 0.00152 |
| Jasmonates Biosynthesis | 0.0023 |
| Pentose phosphate pathway (non-oxidative branch) | 0.01049 |
| Fatty Acid Biosynthesis | 0.01108 |
| Cytokinins-O-glucoside biosynthesis | 0.01451 |
| Fatty Acid Derivatives Biosynthesis | 0.01525 |
| | |
| **Tepal** | |
| Homogalacturonan degradation | 1.62E-08 |
| Carbohydrates Degradation | 2.19E-05 |
| Phenylpropanoid Derivatives Biosynthesis | 7.04E-05 |
| Citrulline biosynthesis | 6.96E-04 |
| Cytokinins-O-glucoside biosynthesis | 0.00157 |
| Cuticular wax biosynthesis | 0.00209 |
| Coumarins Biosynthesis | 0.00269 |
| Xylan Degradation | 0.0136 |
| | |
| **Stigma** | |
| Carbohydrates Degradation | 1.10E-06 |
| Homogalacturonan degradation | 1.71E-04 |
| Cytokinins-O-glucoside biosynthesis | 3.08E-04 |
| Abscisic acid biosynthesis | 0.00182 |
| Carotenoids Degradation | 0.00182 |
| Tetraterpenoids Degradation | 0.00182 |
| Sucrose Degradation | 0.00432 |
| Cytokinins Biosynthesis | 0.00887 |
| Jasmonates Biosynthesis | 0.0097 |
| Plant Hormones Biosynthesis | 0.01351 |
| Selenocysteine Biosynthesis | 0.01405 |
| Generation of precursor metabolites and energy | 0.01417 |
| UDP-glucose biosynthesis (from glucose 6-phosphate) | 0.01431 |
| Fatty Acid Derivatives Biosynthesis | 0.01785 |
| | |

| | |
|---|---|
| **Stamen** | |
| Carbohydrates Degradation | 3.08E-09 |
| Superpathway of sucrose and starch metabolism II (photosynthetic tissue) | 5.65E-05 |
| Cytokinins-O-glucoside biosynthesis | 2.27E-04 |
| Carbohydrates Biosynthesis | 3.65E-04 |
| Homogalacturonan degradation | 5.97E-04 |
| Glycerol and glycerophosphodiester degradation | 6.02E-04 |
| Generation of precursor metabolites and energy | 0.00207 |
| Superpathway of sucrose degradation to pyruvate | 0.00207 |
| Starch degradation I | 0.0021 |
| Sugars Biosynthesis | 0.0048 |
| Cytokinins Biosynthesis | 0.00687 |
| Starch Degradation | 0.0084 |
| Xylan Degradation | 0.0107 |
| Fatty Acid Biosynthesis | 0.01738 |

**Supplementary Table S5** List of primer sequences used for quantitative PCR analysis.

| **Transcript ID** | **Primer sequence** |
|---|---|
| CsTc012245 | GGTCGCACCAGGTCACAAC |
| | CCAAACTCCCTGCCAACTTC |
| CsTc016144 | GACAAGAGACAGAAACAACAAGTAGACA |
| | CGTCGGATAGGATTCAGACACA |
| CsTc018048 | TTTCGTCTGTGTTTCCTTGCA |
| | CGAAAGCTACGTGCTGATAACG |
| CsTc042107 | TCTTGGCGGAGGTGGTTCT |
| | GCTCAGCTTCCGTAACGAAG |
| CsTc048027 | CAGGGAACGGAACTGATACGA |
| | TTGTCGACCGCATCACTGA |
| CsTc054425 | CCGCCGTCTACTACCGTTTC |
| | ATCTCCGTCATTGGCACATCT |
| CsTc001501 | CCACCCGACCCAAGAAGAC |
| | CGATGAGGACCGACCGTTAC |
| CsTc012622 | AAGAGTGAATGCGGTGTGTGAT |
| | GGAGATCGCGGGCAGTAA |
| CsTc016335 | CCGACTCCATCGCCTTTG |
| | TTCACGACGTAGGGAGCTTTC |
| CsTc025071 | CATGTACCGAGAACACGACGAA |
| | TATCCGCCGGCAGATTTC |
| CsTc034793 | CCACTTCCTTCCTCGGTATCG |
| | GGCAATCCCCAGGTTGTTG |
| CsTc038051 | GCTGAGGTTGCCCTAATTGTCT |
| | TGCTGTTGTTGGCATACTCATAGA |
| CsTc056539 | TTGAGGTCGTGCGTCTTGAGTA |
| | GGACCGTGGTCGCTTCCT |
| CsTc070599 | TCTTTGCCTCTCTGGAGTCATG |
| | GGTCTGCGCCGAGTATGC |
| CsTc090197 | ACGCGGTCTCCGGTATCC |
| | TGGGCTAATTTGTGTTATTTGTTTG |
| CsTc102817 | TGGAGAGCAGGACAGCAGAA |
| | TCGATCCATGTTGGTCTCCAA |

**Supplementary Fig. S1** Similarity of *C. sativus* transcripts with proteomes of sequenced plant species (a) and transcriptomes of medicinally important plants (b). Number of *C. sativus* transcripts showing significant (e-value ≤1e-5) similarity with different plants are shown in percentage.

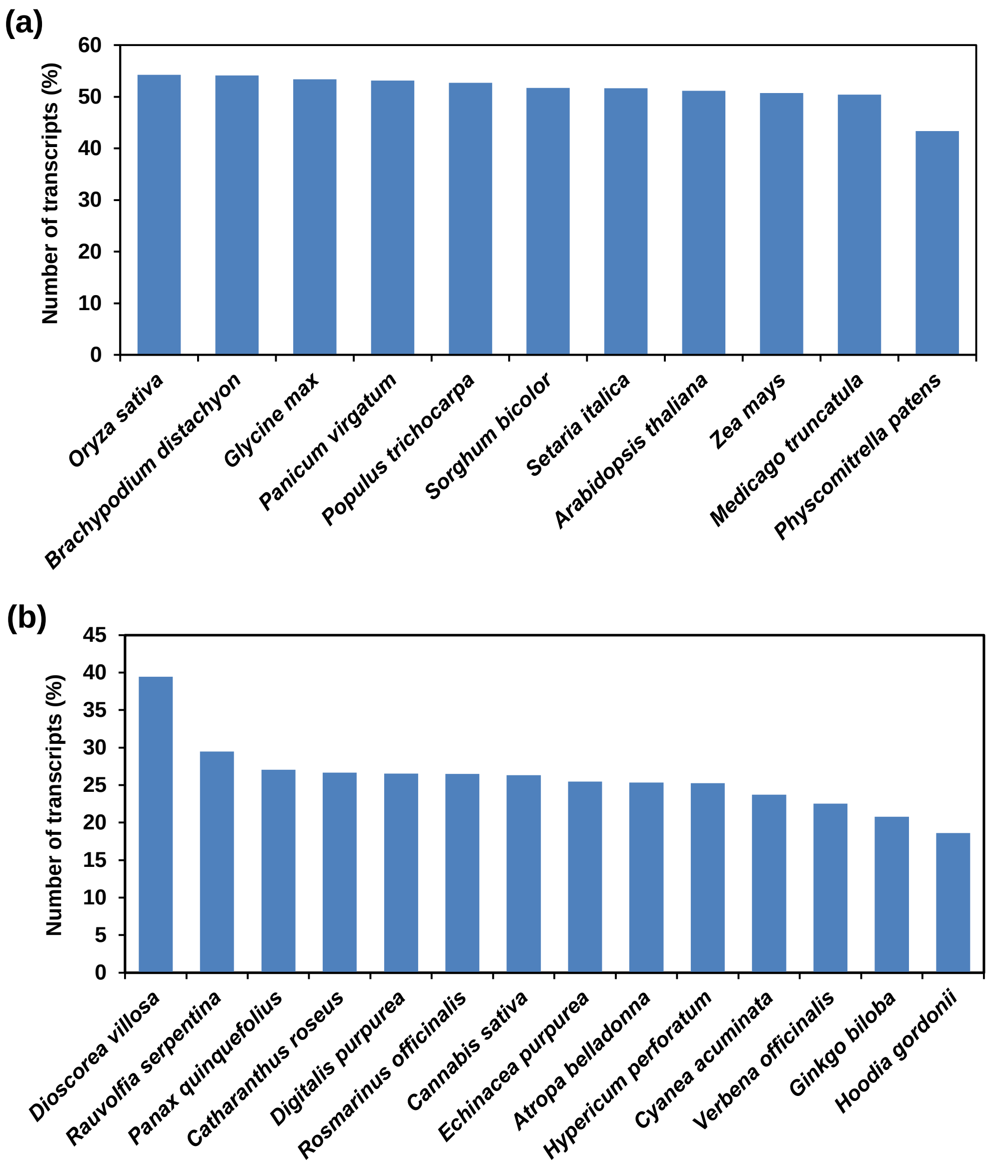

**Supplementary Fig. S2** Different KOG classes represented in the *C. sativus* transcripts.

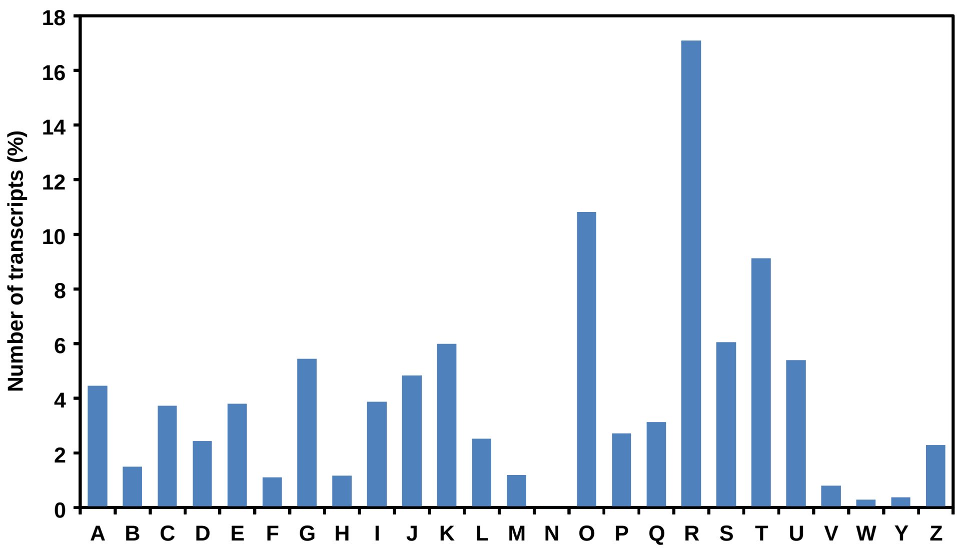


**A** RNA processing and modification
**B** Chromatin structure and dynamics
**C** Energy production and conversion
**D** Cell cycle control, cell division, chromosome partitioning
**E** Amino acid transport and metabolism
**F** Nucleotide transport and metabolism
**G** Carbohydrate transport and metabolism
**H** Coenzyme transport and metabolism
**I** Lipid transport and metabolism
**J** Translation, ribosomal structure and biogenesis
**K** Transcription
**L** Replication, recombination and repair
**M** Cell wall/membrane/envelope biogenesis
**N** Cell motility
**O** Posttranslational modification, protein turnover, chaperones
**P** Inorganic ion transport and metabolism
**Q** Secondary metabolites biosynthesis, transport and catabolism
**R** General function prediction only
**S** Function unknown
**T** Signal transduction mechanisms
**U** Intracellular trafficking, secretion, and vesicular transport
**V** Defense mechanisms
**W** Extracellular structures
**Y** Nuclear structure
**Z** Cytoskeleton

**Supplementary Fig. S3** Heatmap showing the differential expression of selected transcripts involved in terpenoid biosysnthesis. *C. sativus* transcript identifiers are given on the right side. Color scale representing normalized expression values is shown at the bottom.

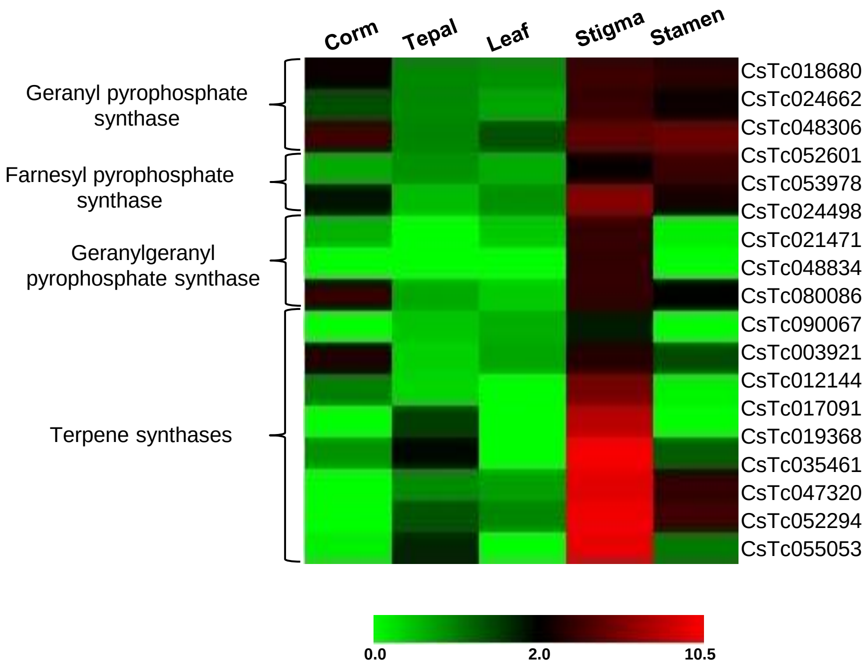

Supplement: Supplementary Information [file srep22456-s1.pdf]
